# Photoacoustic 3-D imaging of polycrystalline microstructure improved with transverse acoustic waves

**DOI:** 10.1016/j.pacs.2021.100286

**Published:** 2021-08-02

**Authors:** Théo Thréard, Elton de Lima Savi, Sergey Avanesyan, Nikolay Chigarev, Zilong Hua, Vincent Tournat, Vitalyi E. Gusev, David H. Hurley, Samuel Raetz

**Affiliations:** a Laboratoire d’Acoustique de l’Université du Mans (LAUM), UMR 6613, Institut d’Acoustique – Graduate School (IA-GS), CNRS, Le Mans Université, France; b Departement of Life and Physical Sciences, Fisk University, Nashville, USA; c Idaho National Laboratory, P.O. Box 1625, Idaho Falls, ID 83415, USA

**Keywords:** Time-domain Brillouin scattering (TDBS), Picosecond acoustic interferometry, Transverse-elastic-waves enhanced contrast, Inclined grain boundary, 3-D imaging, Polycrystalline material, Asynchronous optical sampling, Wavelet synchro-squeezed transform

## Abstract

Non-invasive fast imaging of grain microstructure of polycrystalline ceria with sub-micrometric spatial resolution is performed via time-domain Brillouin scattering. The propagation of a nanoacoustic pulse is monitored down to 8 μm deep in a 30 × 30 μm^2^ area. Grains boundaries are reconstructed in three-dimensions via a two-step processing method, relying on the wavelet synchro-squeezed transform and the alphashape algorithm. Imaging contrast is improved by taking advantage of stronger sensitivity to anisotropy of transverse acoustic waves, compared with longitudinal waves. Utilization of transverse waves in the image processing reveals additional boundaries, confirmed by an electron backscattering diffraction pattern but not discerned using longitudinal waves. A buried inclined interface between differently oriented grains is identified by monitoring changes in amplitude (phase) of the portion of the signal associated with transverse (longitudinal) waves. Estimates of the inclination angle of this interface prove the sensitivity of our laser ultrasonic method to image inclined boundaries.

## 1 Introduction

The photoacoustic (PA) effect, light energy conversion into coherent acoustic waves was discovered in the 19th century [1], immediately followed by the suggestion of its application to spectroscopic analysis of materials. However, not until the 1980s were methodologies such as PA spectroscopy [2] and 2-D PA microscopy [3] developed as reliable materials characterization techniques. Commonly the PA technique is based on the conversion of absorbed laser light into acoustic waves via one or more energy conversion pathways, e.g. thermoelastic, deformation potential, or inverse piezoelectric transduction mechanisms (refer to Ref. [4] for a review). The detection of these generated acoustic waves is accomplished by a variety of approaches, with key examples including inverse piezoelectric transduction and acoustic wave modulation of the phase, amplitude, or propagation direction of optical beams (refer to Refs. [5], [6] for a review). In the case of surface light absorption, spatial resolution can be controlled by the focusing of the laser radiation [7] leading to what is called “super-resolution” [8] and to the micron, i.e. optical, scale imaging [9]. Potentially, the spatial resolution of the PA 2-D imaging could be reduced from “micro” to “nano” by applying near-field optics techniques [10]. The origin of the contrast in imaging via 2-D PA microscopy or nanoscopy is due to variation in the optic, elastic, thermal, and thermoelastic parameters which could influence the conversion process of the pump light into coherent acoustic waves. Because these parameters are tied to crystalline imperfections, PA techniques provide a convenient and non-destructive approach to characterize material microstructure [11]. Most of the imaging techniques involving laser-generated coherent acoustic wave can be grouped according to their physical principles, into one of the three following categories: 1 – laser ultrasonic imaging (LUI), 2 – photoacoustic imaging (PAI) and 3 – time-domain Brillouin scattering (TDBS).

In the first category, imaging is achieved like in traditional pulse-echo ultrasound measurements, by launching coherent acoustic pulses (CAPs) from the sample surface into its volume, and detecting the acoustic waves transmitted/scattered by the sample inhomogeneities, defects, and interfaces [12], [13], [14], [15]. Since the scattered/transmitted acoustic waves are carrying information on the distribution of the acoustic inhomogeneities in the sample, the contrast in LUI is acoustical, and the spatial resolution with depth is controlled by the duration (length) of the laser-generated CAPs. LUI can provide nanometer scale resolution by application of picosecond or femtosecond laser pulses both for the generation and detection of picosecond duration CAPs [16], [17], [18].

In the second category, the local absorption of pump light photons penetrating in the material generates CAPs, profiled with the encoded information on the inhomogeneity of light absorption coefficient spatial distribution. The temporal shape of pulsed-laser-generated PA signal was evidenced in the 1970's to depend on the magnitude of the light absorption coefficient [19], leading the way to measure experimentally the light absorption coefficient spatial distribution from the shape of the detected CAPs (Section 2.2 in Ref. [4]) and applied later for biological tissues study [20], [21]. The photo-generated acoustic waves deliver the encoded information to the detection region near or on the sample surface. PAI performs 3-D imaging at centimeters to micrometers spatial scales [22], [23], [24], [25], [26], [27], [28], [29], [30], [31]. In the acoustic-resolution imaging technique called photoacoustic tomography (PAT), neither the light nor the sound are tightly focused. Different implementations of PAT allow the spatial resolution to be scaled with the desired imaging depth in tissue while a high depth-to-resolution ratio is maintained [32]. PAT can achieve sub-millimeter resolution at depths up to several centimeters [32], [33].

The third category is fundamentally different compared to both LUI and PAI techniques. This approach, known under the names of picosecond ultrasonic interferometry [17], [34], [35], [36] and time-domain (or time-resolved) Brillouin scattering (TDBS) [36], is based on the interaction of probe light with CAPs propagating inside media that are transparent or semi-transparent at the probe wavelength. TDBS and frequency-domain Brillouin scattering differ by the physical origin of the acoustic waves scattering the probe light. Unlike frequency-domain Brillouin scattering, which involves light scattering from incoherent thermal phonons existing in each point of the media and propagating in all directions, TDBS involves light scattering from highly directional CAP beams. The parameters of the CAP beam, such as its frequency spectrum and the directivity pattern, can be controlled like in LUI, via the design of the optoacoustic transducers (OAT) and the choice of the pump light characteristics and optical focusing [4], [36], [37], [38]. Application of ultrashort laser pulses, of picoseconds duration or shorter, provides opportunity to launch coherent acoustic waves in the GHz frequency range. The coherent acoustic waves compose the CAPs, as small as nanometers to sub-micrometers length, that are obtained when the laser pulses are incident on either strongly absorbing materials (like metal or semiconductors with light penetration depths shorter than several tens of nanometers) or just nanometers thick absorbing films/coatings on the transparent substrate. This length scale controls the axial (depth) dimension of the probe light scattering volume, i.e. the dimension along the propagation direction of the CAP. The lateral dimensions of the scattering volume are controlled by the focusing of the pump light, because it determines lateral dimensions of the launched coherent acoustic beam and the angular spectrum of the emitted CAPs [4], [39].

3-D imaging by TDBS is achieved by scanning the co-focused pump and probe laser beams along the surface of a sample. The lateral resolution is controlled by the overlapping pump and probe foci. TDBS was first successfully applied to imaging in transparent media with spatially localized inhomogeneities [40], [41], [42], [43], [44]. Following these seminal studies, the TDBS technique has been extended to 2-D and 3-D imaging of the continuously distributed inhomogeneities in nanoporous materials [45], [46], ion-implanted semiconductors and dielectrics [47], [48], [49], texture in polycrystalline materials [[50], [51], [52]] or inside vegetable and animal cells [53], [54], [55], and temperature profiles in liquids [56]. All these experiments confirmed that, when the CAP is reflected/transmitted by an interface between two transparent media, a TDBS signal can change both in periodicity and amplitude. Changes in periodicity are due to different acoustical and different optical properties in each medium. Amplitude changes are additionally due to different acousto-optical parameters in each medium as well as acoustical and optical impedance mismatches across interfaces. The characterization of the spatial orientation of plane interfaces or, more generally, imaging of complex non-plane interfaces by TDBS in the bulk of a medium, has emerged quite recently in relation to polycrystalline materials with application in the energy industry [57], [58], [59], following earlier experiments where the surface acoustic waves generated and detected by ultrafast lasers were applied for the imaging of grain boundaries on the surface of a medium [60].

In Ref. [57], a 2-D image of a subsurface grain boundary in a polycrystalline UO_2_ sample was obtained due to the variations of both the frequency and the amplitude of a monochromatic Brillouin oscillation, when CAP was crossing the boundary. In [58], the 3-D TDBS imaging was applied to the evaluation of the elastic constants of CeO_2_ and determination of the crystallographic orientation of the grains on the surface of the sample. It was demonstrated that TDBS imaging with a quasi-transverse acoustic (TA) pulse can be more sensitive to grain orientation than TDBS imaging with quasi-longitudinal acoustic (LA) pulse. 3-D orientation of the subsurface boundary by TDBS imaging with LA mode was also reported. Finally, it has been demonstrated very recently [59] that complete orientation of the surface grains can be obtained by measuring how the polarization of the probe beam influences the detected signal amplitude in the TDBS imaging. These recent studies on imaging grain microstructure with TDBS lead to the ability of 3-D imaging of grains and grains boundary in large volumes of polycrystalline materials. Yet, the complete 3-D imaging in a large volume (about 7200 μm^3^ here) remains to be addressed, due to the current lack of a dedicated and robust signal processing analysis routine and image reconstruction procedures. In this manuscript we present a method for efficient and complete 3-D image reconstruction of grain microstructure in polycrystalline CeO_2_.

The CeO_2_ sample and the experimental scanning system are described in Section 2. TDBS is introduced and the step-by-step processing of the TDBS signals is detailed, from the photodiode measurements to the 3-D imaging results. In Section 3, the 3-D imaging results are presented. The contribution of the transverse acoustic waves reveals “new” grains via higher contrast. An inclined interface is uncovered, buried below the free surface of the sample, and its angle of inclination with respect to the surface is estimated. Limitations of the method to untangle the different contributions to the TDBS signal at the interface are discussed. The conclusions are stated in Section 4.

## 2 Materials and methods

### 2.1 Sample and experimental setup descriptions

The sample studied is a polycrystalline cerium dioxide (or ceria) CeO_2_, with an optical refractive index *n* = 2.37 at probe wavelength *λ* = 535 nm, estimated from the measurements (in good agreement with [61], see appendix for details). The sample was annealed at 1650 °C to promote grain growth (see Ref. [58] and supplementary materials for details), the average grain size being ∼20 μm. A focused ion beam was used to put down fiducial marks. The crystallite orientation at the surface of the sample was obtained using electron backscatter diffraction. The fiducial marks are visible optically and in the EBSD micrograph and are used to locate specific grains. A 20 nm-thick gold layer was coated to serve as an opto-acoustic transducer (OAT). Ceria, which is optically isotropic and elastically anisotropic because its crystalline structure is cubic, provides a model material for demonstrating enhanced imaging using transverse acoustic waves, without the complication of optical birefringence. In the case of birefringent materials, i.e. optically anisotropic crystals, polarimetry measurements would be required to distinguish ordinary from extraordinary light contributions to the TDBS signal. Moreover, refraction of the probe light at the interface between differently oriented grains would modify the scattering efficiency in the deepest grain, while in the presented case there is no refraction of light at the interface.

**Fig. 1:**
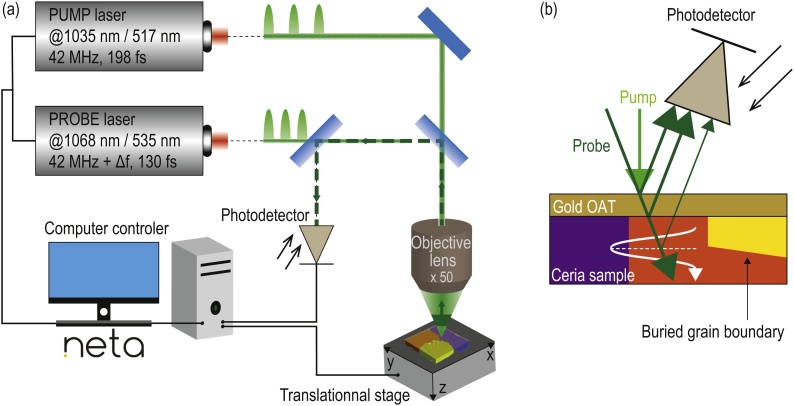
(a) Schematic of the experimental TDBS imaging system (JAX-M1) commercialized by NETA (France). (b) Schematic of the probe beam reflections in TDBS (reflection configuration): two stationary reflections occur at the OAT interfaces, while a third weaker reflection occurs at different depth along the propagating path of the acoustic pulse. A buried inclined boundary between two grains is represented in the right part of the polycrystal's schematic.

Measurements of the transient reflectivity signals were done using a pump–probe Asynchronous Optical Sampling (ASOPS) system: the JAX-M1 commercialized by NETA [62], [63], [64] [Fig. 1(a)]. The setup is composed of two Yb:KYW mode-locked laser cavities, emitting femtosecond laser pulses. The pulse delay between the two lasers is accomplished electronically by changing the pulse repetition rate of the follower laser keeping the leader laser repetition rate fixed. The difference in the frequency of repetition between the two lasers, or the beat frequency, is set to Δ*f* = 500 Hz. Both pump and probe beams are focused, using a 50× microscope objective lens, on the free surface of the gold coated sample. Only the second harmonics of the laser sources are used. The wavelength of the probe (leader laser) is *λ*_probe_ = 535 nm, while that of the pump (follower laser) is *λ*_pump_ = 517 nm. To monitor the relative optical reflectivity change Δ*R*/*R* caused by the propagating CAP, the backscattered probe light is directed to a photodiode where it interferes with static reflections of the probe beam on the sample surfaces [Fig. 1(b)]. The automatic scan and signal acquisitions are realized using NETA's built-in software. More details on the ASOPS-based setup can be found in [62]. The sample being placed on a displacement stage, and due to the speed of a single-point measurement, a scan lasting less than five hours is realized on a 30 × 30 μm^2^ area, with a lateral resolution of 0.469 μm. Probed depth reaches 8 μm in the most favorable cases, with an axial (i.e. along the depth) resolution of about 0.75 μm (more details on depth of imaging can be found in the supplementary materials).

### 2.2 Time-domain Brillouin scattering (TDBS)

The TDBS method aims to study materials, transparent or partially transparent to the wavelength of the probe laser beam *λ*_probe_, by monitoring the propagation of a coherent acoustic pulse (CAP), generated in the OAT by the pump laser pulse. The thermo-elastic mechanism is responsible for acoustic wave generation: the pump laser pulse is focused on the surface of the OAT, inducing a fast temperature rise (heating) hence deformation of the material by thermo-elastic stresses. This process generates acoustic waves that can be considered plane (large lateral-distribution-to-acoustic-pulse-length ratio) and that propagate in the OAT, normal to the free surface, until reaching the interface OAT/CeO_2_ where the acoustic waves are transmitted in ceria. In our case, the time-delayed probe laser pulse is focused on the same side as the pump laser.

In the TDBS imaging experiments reported here, a portion of the probe light reflected from the propagating CAP is measured via heterodyning with the portion of the probe light reflected from the OAT [Fig. 1(b)]. This Brillouin scattering of probe light contributes to the total variations of the transient reflectivity and gives rise to an oscillating component in time, due to the interferences with stationary scattered light. Periodicities of these oscillations are related to the phase shifts acquired by the probe light when scattered from the acoustic pulses travelling in the media. For a probe beam parallel to the direction of the acoustic pulse propagation, the frequency *f*_B_ of the Brillouin oscillations is linked to the velocity v of the propagating pulse and the optical refractive index *n* of the material at probe wavelength by:

(1) $$f_{B}=\frac{2nv}{\lambda_{\mathrm{probe}}}.$$

When depth profiling with TDBS, the information from different spatial positions along the acoustic pulse propagation path inside the sample is obtained. The phase of the probe light scattered by the CAP contains the information on the product of the local optical refractive index by the local acoustic velocity [17], [34], [36], [45], [46], [65]. The amplitude of the scattered light also contains local information on the components of the photo-elastic tensor, which determines the strength of acousto-optic interaction [36], [46], [47], [66], [67].

Three acoustic quasi-modes can be launched in the elastically anisotropic ceria: a quasi-longitudinal acoustic (LA), a “slow” transverse acoustic mode (sTA) and a “fast” transverse acoustic mode (fTA). Generation efficiency of transverse acoustic waves has been recently studied in Ref. [68]. By considering multiple crystallite orientations, an estimate of the Brillouin frequency (BF) range can be obtained for each acoustic mode (Table 1). More details on the computation of this BF intervals are given in the supplementary materials. In the following, transverse modes with frequencies falling in the common BF interval [26.79, 37.97] GHz are labeled as TA, while modes above this interval can be unambiguously labeled as fTA modes.

**Table 1:** Expected Brillouin frequency (BF) intervals to be detected with TDBS in the case of cubic ceria CeO_2_, for each acoustic quasi-modes, using elastic properties and density from [58], [59].

Acoustic mode	Frequency interval (GHz)
LA	[58.77, 70.02]
sTA	[26.79, 37.97]
fTA	[26.79, 42.48]

### 2.3 On the processing of transient reflectivity signals

The raw transient reflectivity signals are processed as follows. First they are filtered in order to isolate the acoustic contribution (oscillating part of the signal) from the total transient reflectivity. A three-steps procedure starts by denoising the raw transient reflectivity signals with a 48th-order finite impulse response (FIR) bandpass filter with a passband in the range [25, 75] GHz tuned to the expected BF of Table 1. The initial “electronic” peak in the signals, corresponding to the overlapping time of both pump and probe laser pulses, is removed since it is not related to the acoustic contribution. The first 14.3 ps are cut to remove the electronic response near *t* = 0 ns, meaning that the first nanometers in contact with the gold nanolayer (OAT) are not used (i.e. considered homogeneous) for the depth vector reconstruction. Finally, to estimate and subtract the remaining contribution of the thermal processes, we apply a local linear regression method (LOESS from MATLAB) over 500 points (0.24 ns) to the filtered peak-free signal. This treatment is applied to all the 3600 signals of the scan presented in this paper. The signals coming out of these three steps of filtering, referred to as the “acoustic signal”, are considered to contain exclusively the acoustic contribution of the signals and are further processed as discussed in the following.

### 2.4 On the processing of the acoustic signals: slicing

The slicing of each acoustic signal, to study the evolution of their frequency content as a function of time, is performed via the wavelet synchro-squeezed transform (WSST) [69], [70]. This tool aims to narrow the time-frequency representation obtained with the wavelet transform by re-allocating the spectrum energy along the frequency axis only. Such WSST tools are used, in combination with ridge extraction, to reconstruct the temporal behavior in time of a superposition of AM/FM modes, in a noisy signal [71], [72]. Applications to paleoclimate datas [73] showed the robustness of this method, now extended to a variety of signal decomposition problems [74], [75]. Performing a ridge extraction, extraction of the maximum-energy time-frequency ridge of the spectrum, offers the possibility to evaluate with precision the BF of the acoustic mode studied, for each time step. The elegance of the WSST is found in its capacity to offer a clear enough spectrum so that 3 ridges extraction can be performed, as long as these ridges are sufficiently separated in frequency (refer to Definition 3.2 on intrinsic mode components separation in Ref. [69] for further details). The ridge is extracted along the full measurement time, but only the points of the ridge with energy amplitude over a −68 dB threshold value are kept.

**Fig. 2:**
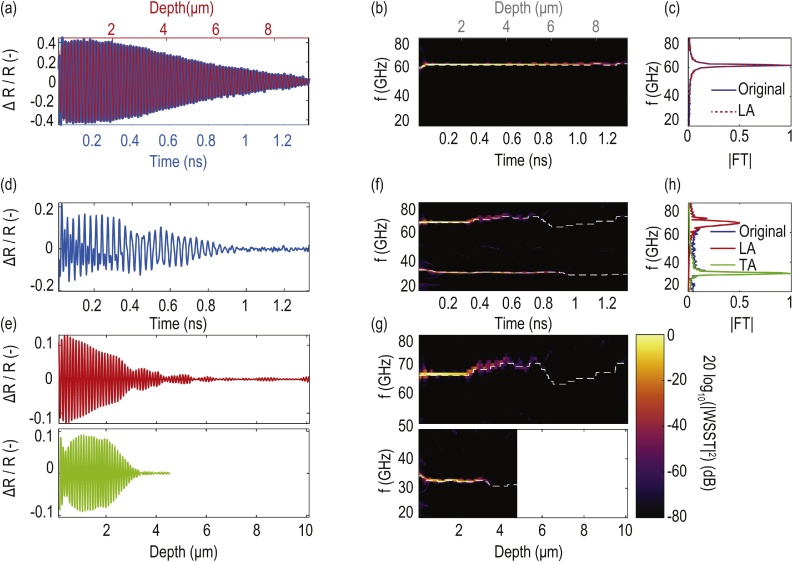
Analysis of two typical acoustic signals of the scan (a–c) and (d–h). (a) Acoustic signal (blue signal and lower *x*-axis) and reconstructed LA contribution (red signal and upper *x*-axis) of the signal located at coordinates (25.79 μm, 23.45 μm) in the scan. (b) Associated WSST-based spectrogram and extracted ridge (dashed white line on the spectrogram). (c) Associated Fourier transform (FT) of the full signals of (a). (d) Acoustic signal located at coordinates (9.38 μm, 11.72 μm). (e) Reconstructed LA (red, upper) and TA (green, lower) contributions to the acoustic signal (d). (f) Associated WSST-based spectrogram and ridges (white dashed lines on the spectrogram) of the acoustic signal (d). (g) Associated WSST-based spectrograms and ridges (white dashed lines on the spectrogram) of the reconstructed LA (red) and TA (green) contributions (e), zoomed on the frequency of interest. (h) Associated Fourier Transform (FT) of the full signals (d) and (e) affiliated by their colors. The depth vectors are reconstructed from the instantaneous frequency of the ridge extracted from the associated acoustic mode and Eq. (1), explaining the “downsizing”of the slower TA mode in the lower part of (e) and (g). (For interpretation of the references to color in this figure legend, the reader is referred to the web version of this article.)

For example, the reconstruction of a single-frequency signal is depicted in Fig. 2(a). The acoustic signal measured at the position (*x*, *y*) = (25.79 μm, 23.45 μm) in the scan is represented in blue (with associated *x*-axis in blue). The signal in red (with associated *x*-axis in red) correspond to the contribution of the LA mode to the acoustic signal. The temporal behavior of LA mode contribution is reconstructed from the ridge extracted in the spectrogram obtained with the WSST [Fig. 2(b)] by applying the inverse WSST (IWSST). The LA mode contribution is plotted with respect to depth, the depth vector being reconstructed with Eq. (1) and the instantaneous frequency, i.e. local velocity, of the extracted ridge. The Fourier Transform (FT) of both full signals [Fig. 2(a)] confirms the presence of a single-frequency component, corresponding to the monitoring of a long-lasting LA mode. Another signal, located at the position (*x*, *y*) = (9.38 μm, 11.72 μm), is given in Fig. 2(d). This signal contains two distinctive frequencies, confirmed by its WSST spectrum [Fig. 2(f)], sign that two different acoustic waves are monitored. The contributions to the acoustic signal associated to the two modes are plotted with respect to the reconstructed depth in 2(e), the contribution of the LA mode being in the upper part in red, and the one of the TA mode in the lower part in green. The depth vector of both modes are reconstructed thanks to Eq. (1) and their associated instantaneous frequency in the extracted ridges of their WSST spectra [Fig. 2(g)]. Velocities of the TA waves are predicted to be nearly two times slower than the velocity of the LA waves, explaining the two-times shorter depth of reconstruction of the TA mode (lower part of Fig. 2(e) and (g)). Finally, the FT [Fig. 2(h)] of all the signals [Fig. 2(d) and (e)] confirms the presence of the two modes and their good frequency separation during the reconstruction.

Knowledge of the BF of the acoustic mode for each time step allows the conversion from time to depth using Eq. (1). For each time step, the acoustic wave velocity associated with the extracted BF allows an estimate of the travelled depth since the previous time-step. The time-frequency ridge extracted from one acoustic mode of one signal becomes a depth(*z*-axis)-frequency ridge. Assembling all the depth frequency ridges of the 3600 signals of the scan, for one of the three possible acoustic quasi-mode, leads to a 4-D “sliced” representation of the complete set of collected data.

### 2.5 On the processing of the acoustic data: shaping

**Fig. 3:**
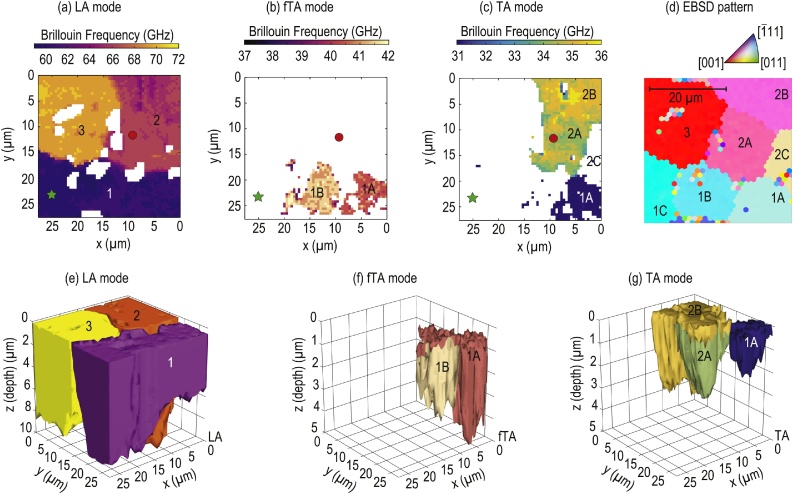
Imaging results: (a–c) upper view (free surface of the sample) of the slicing output for each BF interval [Table 1], (d) electron backscattering diffraction (EBSD) pattern of the scanned area, and (e–g) 3-D alphashape representations (e–g) of the associated (color and order) BF interval (a–c). Showing the upper view of the 4D “stacked” representation from the slicing processing step of the (a) LA, (b) fTA and (c) TA modes (colors associated to the BF) offers the possibility to appreciate the good agreement between the first slices and the EBSD pattern (d). The positions of the previously analyzed signals are represented by a green star [Fig. 2(a)–(c)] and red circle [Fig. 2(d)–(h)]. The alphashape results evidence the reconstitution of 3 grains (1/purple, 2/orange and 3/yellow) with the LA mode (e), 2 grains (1A/salmon-red and 1B/beige) with the fTA mode (f) and 3 grains (1A/blue, 2A/green and 2B/yellow) with the TA mode (g). One can identify how TA modes allow to identify smaller grains from bigger homogeneous ones imaged by LA modes. (For interpretation of the references to color in this figure legend, the reader is referred to the web version of this article.)

From the 4-D output of the slicing step (Section 2.4), one voxel is defined by its BF and its 3-D location (*x*, *y*, *z*). A grain is thus defined by a group of adjacent voxels with common BF. To form a grain, it is assumed that the corresponding group of adjacent voxels should be composed of at least 5% of the total voxels of the 4-D stacked representation for each mode. The 5% threshold allows to denoise the final 4-D representation of grains, with the drawback of removing small grains from the image. Typically, in the case of LA mode, the total volume occupied by the voxels after thresholding the ridge extraction in the slicing step reduces from 7200 μm^3^ to about 4800 μm^3^ (Table 2). This means that grains with a volume less than approximately 240 μm^3^ are neglected in the LA reconstruction due to the 5% threshold. Note that in the case of the fTA mode, the volume occupied by the voxels after thresholding is 450 μm^3^, thus the minimal volume of a grain in that case is of about 23 μm^3^, while in the case of TA mode, the volume occupied by the voxels is 680 μm^3^, hence the minimal grain volume is of about 34 μm^3^. These estimates seems to be acceptable in our case since grains are expected to have larger volumes, the smallest grain being depicted by the blue grain reconstructed by TA detection in Fig. 3(g). The number of grains, and the associated BF intervals bounding the gathered voxels, are estimated via a segmentation method inspired from Otsu's work (details can be found in supplementary materials) [76]. This method identifies each cloud of voxels containing common BF that are used as an input to the alphashape algorithm [77], [78]. This algorithm relies on a constraining parameter 1/*α*, linked to the radius of a sphere, to shape convex and non-convex envelope of the cloud of voxels. Hence an infinite radius leads to the convex hull of the cloud of voxels, while a zero radius gives the cloud of voxels itself (no envelope). The different envelopes obtained between these two particular cases are named the family of alphashapes of the cloud of voxels. In the following analysis, the radius is set as twice the lateral spatial resolution of the scan, thus 1/*α* = 1 μm. The shaping of each cloud of voxels containing common BF-segmented interval offers a volumetric representation of all the boundaries of a single grain (see Fig. 3 and related discussions in the following section).

**Table 2:** Volume estimates of the alphashape reconstructed grains.

*LA grains*	Volume (±4.2 μm^3^)

1 (purple)	1530
2 (orange)	1336
3 (yellow)	1901

*fTA grains*	Volume (±4.2 μm^3^)

1A (salmon-red)	264
1B (beige)	186

*TA grains*	Volume (±4.2 μm^3^)

1A (blue)	97
2A (green)	237
2B (yellow)	344

## 3 Results and discussion

### 3.1 3D imaging enhanced with transverse waves

The signal shown in Fig. 2(a), which corresponds to a grain with no subsurface boundaries within the interrogation volume, is used to assess the maximal depth of imaging of our system. This depth is ∼8 μm, attributed to the TDBS imaging coherence penetration depth of the probe laser (details in supplementary materials). The signal shown in Fig. 2(d) offers insight into multi-component signals with BF in the LA mode interval as well as in the TA mode interval. The reconstruction of the temporal behavior [Fig. 2(e)], and its change with depth, associated with both acoustic modes gives the opportunity to extract complementary information used for the imaging. The boundary between two differently-oriented grains is evidenced by the two modes via two different physical mechanisms. First, the LA mode is subject to an upshifting of its BF [Fig. 2(g)] that can be attributed, following Eq. (1), to the increase of the LA mode velocity due to its transmission to a differently-oriented grain. In parallel, the TA mode amplitude suddenly decreases to the noise level [Fig. 2(e) and (g)] at the same corresponding depth (∼3 μm) at which the BF of the LA mode is upshifted. This sudden drop in amplitude of the TA mode after transmission into the second, subsurface grain is the signature of: either large angle, off-axis refraction of the TA mode such that the interaction with the probe is vanishingly small, or small photoelastic coupling in the new grain with different orientation. In the specific case of the signal (d) and (e) in Fig. 2(b), the amplitude drop is associated with the latter case, because the deepest grain has a [0 0 1] orientation and such an orientation preclude TA mode detection.

The 4-D stacked, or slice-by-slice, representations obtained as an output of the slicing offers a mode-by-mode view on the information of each extracted ridge, analogous to a loaf of sliced bread. Looking to such representation from the top gives a view on the first few nanometers of the sample, i.e. the gold-coated surface of the ceria sample [Fig. 3(a)–(c)]. One can identify the different grains from their associated BF (colors), white zones being attributed to signals with no exploitable results (photodiode saturation and or amplitude of the associated WSST ridge below the −68 dB threshold defined previously). These first layers of the sample reconstructed can be directly compared to the EBSD pattern of the scanned area of the sample. The localizations of the two signals analyzed in Fig. 2 are represented, on these top views, as a green star [Fig. 2(a)–(c)] and a red circle [Fig. 2(d)–(h)]. Imaging results [Fig. 3(e)–(g)], output of the shaping, reconstruct the 3-D boundaries of the grains detected by each modes: the LA mode [Fig. 3(e)] brings out three main grains (1/purple, 2/orange and 3/yellow) while fTA/TA modes interval identifies smaller ones insides these main LA grains. The fTA mode [Fig. 3(f)] deconstruct the 1/purple grain into three smaller ones: 1A/salmon-red, 1B/beige and a third one, 1C, that is evidenced thanks to the absence of detection of the fTA mode. The TA mode [Fig. 3(g)], similarly, deconstruct the 2/orange grain into three smaller ones: 2A/green, 2B/yellow and a third one, 2C, depicted by the absence of TA mode in it. Moreover, part of the 1A grain is reconstructed also with the TA mode as 1A/blue [Fig. 3(g)]. This grain presents the peculiarity of being imaged by the three acoustic quasi-modes: LA (a), (e), fTA (b), (f) and sTA (c), (g) [Fig. 3]. The *z*-axis (depth) scales are enlarged for better visualization, the ratio between the *z*-axis and the *x*- and *y*-axes are 1/6 in Fig. 3(e) and 1/12 in Fig. 3(f) and (g). Concerning the axial (depth) resolution, its estimation depends on the acoustic quasi-mode studied, since it is linked to the temporal resolution of the corresponding wavelet in the WSST. However, the acoustic velocity (and hence BF) ratio between the TA waves and the LA ones being close to 2 leads to approximately 0.75 μm resolution for both a LA wave detected at 64 GHz and a TA wave detected at 34 GHz, while the temporal resolution of the wavelet is such that it includes at least 6 full oscillations of the associated BF wavelength.

The use of the alphashape algorithm offers the possibility to estimate the volume of the reconstructed grains (Table 2). The uncertainties are obtained using the definition of the alphashape: the radius of the sphere used to shape our voxels clouds is 1/*α* = 1 μm thus the volume uncertainty is linked to the volume of the corresponding sphere of radius 1 μm.

When detected, the stronger sensitivity to anisotropy of the TA modes offers an imaging contrast that was not achieved by monitoring the LA mode, as evidenced in the upper part of Fig. 3(a)–(c). Looking to the first slices of the processing, the LA mode results in Fig. 3(a) seems to indicate the presence of three main grains in the scanned area, at the interface gold OAT/CeO_2_. The same processing applied to the fTA [Fig. 3(b)] and TA [Fig. 3(c)] modes leads to a new interpretation of the LA mode results, highlighting the presence of smaller grains: the LA purple grain in Fig. 3(a) is in fact composed of three grains, two of them being evidenced by the fTA mode results [Fig. 3(b)], and one of the two being also imaged by monitoring the sTA mode propagation [blue grain in Fig. 3(c)]. In this specific grain, all the three possible acoustic quasi-modes are monitored, offering the possibility to estimate its crystalline orientation in the laboratory frame [79], [80].

Another example of the imaging enhancement permitted by TA modes detection is given in Fig. 3(c) where two grains, that were originally imaged as one grain on the LA mode results [Fig. 3(a)], with close, but different, BF of a TA mode are pictured in green and yellow. One can also deduce the presence of a third grain, 2C, located in the scan interval *x* ∈ [0, 5] μm and *y* ∈ [10, 20] μm, not detected in the TA representation (white space in Fig. 3(c)), and included in the orange grain on the LA mode results [Fig. 3(a)]. Finally, thanks to the additional information obtained with the TA modes, a complete view of the ceria polycrystal is achieved by TDBS and is in a very good agreement with the EBSD pattern measured on the surface of the sample [Fig. 3(d)]. The imaging contrast provided by the TA modes leads to the detection of the same number of grains as the EBSD pattern. The extension of these observations from the OAT vicinity to the depth of the grains, with the alphashape 3-D results shown in Fig. 3(e)–(g), presents the evolution of the grains boundaries with depth. The 3-D representation obtained with the LA mode sheds some light on a small grain appearing only in the depth of the sample (from 6 to 8 μm deep), below the violet one, with a BF close to the interval of the orange grain hence colored in orange in Fig. 3(e). Such deep transition from one grain to another, not visible using electron microscopy without destructive serial section, highlights the capacity of the TDBS technique to image grain microstructure nondestructively in 3D.

### 3.2 Buried interface inclination estimation

The 3-D alphashapes results appear as a suitable representation to locate buried inclined interfaces between differently oriented grains. One such surface is found to be between the orange (upper) and yellow (lower) grains of the LA mode alphashape [Fig. 3(e)], located in the center of the scanned area and between 2 and 3 μm deep. Evidenced by both the LA and one TA modes, the inclination of this interface can be assessed by following the behavior of the TDBS signals in the vicinity of this area, one of the signals being represented in Fig. 2(d). As already mentioned, such signals are sensitive to the crossing of the interface by the acoustic pulse accordingly evidenced by the frequency shifting of the LA mode and the amplitude drop to the noise level of the TA mode. The situation when a CAP is incident on an inclined boundary between differently oriented grains leads to the reflection and refraction of the CAP (and not of the probe light due to the optical isotropy of ceria), and could lead to the mode-conversion of the incident CAP into up to three reflected and three transmitted CAPs with different polarizations [81]. For the interface inclinations observed in this manuscript, the propagation directions of the reflected CAPs deviate more importantly than the directions of the transmitted CAPs, in comparison with the most efficient detection direction, i.e. the propagation direction of the probe light. The reason of these detection reductions is linked to the efficiency of the light scattering by the CAP (acousto-optic interaction), being reduced due to the deviation from the momentum conservation law of the photon-phonon interaction. This deviation from the optimal scattering geometry results in the weakening of the scattering of the probe light and thus of the heterodyne detection, causing a diminution of the amplitude of the TDBS signal. Thus, an abrupt decrease to the noise level of the contribution to the TDBS signal from the reflected CAP is owing to its deviation from the propagation direction of the probe light (which is about 2*θ* in weakly elastically anisotropic crystals like ceria), while the transmitted CAP contribution is “only” weakened for the discussed small angles. Considering small angles results in significantly smaller refraction angles of the CAP in comparison with the reflection angles. Because of the low elastic anisotropy of ceria, its Zener ratio being *α*_*_r_*_ = 2*C*_44_/(*C*_11_ − *C*_12_) = 0.4 [58], the contribution to the TDBS signal of the mode-converted transmitted CAP is considered confined to the contribution of the transmitted LA mode only. On one hand, low anisotropy could lead to weak mode-conversion, and, on another hand, the resulting transmitted TA waves would be composed in minority of a longitudinal component, which is the component efficiently scattering the probe light for the heterodyne detection, and in majority of transversal components, efficiently depolarizing the probe light, i.e. precluding it for being detected. In conclusion, for the experiments reported in this manuscript, the contributions to the TDBS signal of the reflected and the mode-converted transmitted TA waves are considered to be too small to be accounted for, even though they are existing.

**Fig. 4:**
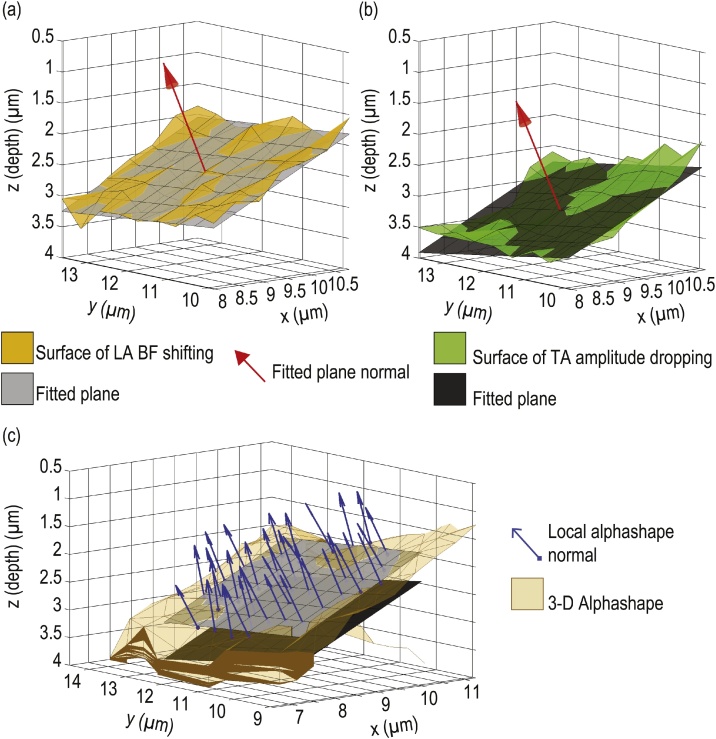
Inclined buried interface analysis. (a) Surface of frequency shifting of the LA mode (orange surface) and best (least mean square) fitted plane (grey plane). (b) Surface of TA mode's ridge amplitude (in the WSST spectrogram) drop below the −68 dB threshold (green surface) and best (least mean square) fitted plane (black plane). The red arrows depict the outward normal of the fitted plane used to estimate the mean inclination angle of the surface. (c) Comparison of the alphashape LA mode results (yellow triangles mesh) and the two fitted planes of (a) and (b). The blue arrows, with a square base, highlights the local normals used to estimate the averaged angle over the alphashape region. (For interpretation of the references to color in this figure legend, the reader is referred to the web version of this article.)

A representation of the buried surface at which the frequency shifting in the LA occurs is shown in Fig. 4(a). Similarly, a representation of the buried surface at which the amplitude drops for the TA mode is shown in Fig. 4(b). Note that the absolute depth at which the buried surface is found from LA and TA mode are slightly shifted, due to the blind zone in the first ps of the temporal signals, while this is not influencing the inclination angle recovery. Fitting the least-mean square plane to each surface gives access to a plane from which the mean inclination angle (with respect to the gold OAT, z = 0 μm plane) is estimated via its outward normal. Results obtained from the frequency shifting of the LA mode [Fig. 4(a)] gives 23.3°, and from the amplitude drop of the TA mode [Fig. 4(b)] gives 23.2°. Comparisons of the alphashape result of the lower grain of the interface with the fitted planes shown in Fig. 4(a) (LA mode) and Fig. 4(b) (TA mode) are shown in Fig. 4(c). A good agreement between the inclinations of the fitted plane and the actual alphashape result is shown. The local normals of the alphashape's triangle mesh of the same area are determined (blue arrows) and the averaged angle between these local normals and the gold OAT gives a 23.8° inclined interface. Hence a good agreement between the fitted planes to the LA and TA modes surfaces, and the alphashape reconstruction, allows estimation of the inclination of this interface to be of about 23.5 ± 0.5° with respect to the z = 0 μm plane.

## 4 Conclusions

A method to reconstruct the 3-D grains boundaries of a polycrystalline ceria (CeO_2_) sample, down to 8 μm depth, from opto-acousto-optic measurements, has been implemented. This technique provides opportunity to recreate an accurate 3-D image of grains and their respective boundaries. Time frequency tools are developed for the reconstruction of the temporal contribution to the measured signal of each possible acoustic mode independently. Grain boundary identification has been enhanced by monitoring of the acoustic quasi-transverse modes, highlighting boundaries that were not visible via quasi-longitudinal mode detection only. This new signal processing method opens the possibility of non-destructive, *in situ* visualization of grain shape and its evolution beneath the surface. The angle of a buried inclined interface between two differently oriented grains is estimated to be about 23.5°. Extraction of such fundamental information (size and orientation) has application in the characterization of in-situ/real-time microstructure evolution in extreme environments including high temperature and pressure, high magnetic and electric fields, and harsh irradiation environments.

## Authors’ contribution

S.R., S.A., N.C., V.T., D.H.H. and V.E.G. designed the research, D.H.H. and Z.H. prepared the sample, E.L.S., S.R., S.A., N.C. and D.H.H. contributed to the experiments, T.T. and S.R. contributed to signal processing, T.T., S.R., N.C., V.T., D.H.H. and V.E.G. analyzed and interpreted the experimental observations, T.T., S.R., D.H.H. and V.E.G. wrote the manuscript. All authors reviewed the manuscript.

## Declaration of Competing Interest

The authors declare that they have no known competing financial interests or personal relationships that could have appeared to influence the work reported in this paper.
