# Supplementary material for: Photoacoustic 3-D imaging of polycrystalline microstructure improved with transverse acoustic waves
Source: Photoacoustics. 2021 Aug 2;23:100286. doi: 10.1016/j.pacs.2021.100286 (PMC8371231; doi:10.1016/j.pacs.2021.100286)
Supplement: Supplementary file 1 [file mmc1.pdf]

Supplementary Materials

Supplementary materials on the 3-D slowness surfaces of ceria (CeO_2) and optical refractive index estimation

Up to three acoustic quasi-modes can be generated in the cubic polycrystal CeO_2. The determination of the associated BF intervals requires the estimation of the fastest and slowest acoustic velocities for each quasi mode. To achieve this, the Christoffel equation is solved and the velocities v associated to any direction are given by the roots of the secular equation (following Ref. [81] - Chapter 4 notation):

$$|\Gamma_{\mathrm{ij}} - \rho v^2 \delta_{\mathrm{il}}| = 0, \tag{2}$$

with Γ_{ij} Christoffel's tensor, ρ ceria's density and δ_{ij} the Kronecker symbol. The elastic constants ($C_{11} = 451$ GPa, $C_{12} = 119$ GPa and $C_{44} = 66$ GPa) and the density $\rho = 7.22\ \mathrm{g\,cm^{-3}}$ are given in Ref. [57]. The 3-D slowness (inverse of velocity) surfaces of the three quasi-modes are plotted in Fig. 5 which shows how much faster the LA waves (inside surface - red) are compared to both TA ones. The slowness surfaces also clearly depict that both TA modes are more sensitive to changes in the propagation direction with respect to the crystal principal axes than the LA mode. The results of table 1 are obtained by applying Eq. 1 to the fastest and slowest velocities of each mode, leading to the presented estimates of the BF intervals.

Determination of the optical refractive index n, of our ceria sample, to the probe wavelength $\lambda = 535$ nm is made possible thanks to the detection of the three acoustic quasi mode in a specific grain of the area scanned, located in the spatial interval x = [0, 5] µm, y = [20, 25] µm, 1A in Fig. 3. Indeed, from the slowness surfaces, it is possible to represent the two "surfaces of shear velocity ratio to LA" for the two TA modes [Fig. 6]. In Eq. 1, λ and n are fixed, thus the ratio of velocities is equal to the ratios of the BFs. These two ratios are used to locate, in the two "surfaces of shear velocity ratio to LA" the possible orientations and thus the associated acoustic velocities. Finally, by isolating n in Eq. 1, the optical refractive index can be estimated for the probe wavelength. Table 3 gives the values obtained with their associated uncertainties given by the frequency resolution of the ridge extracted in the WSST, the BF interval being associated to the corresponding frequency bin in the histogram of the segmentation method (see next section for more explanations).

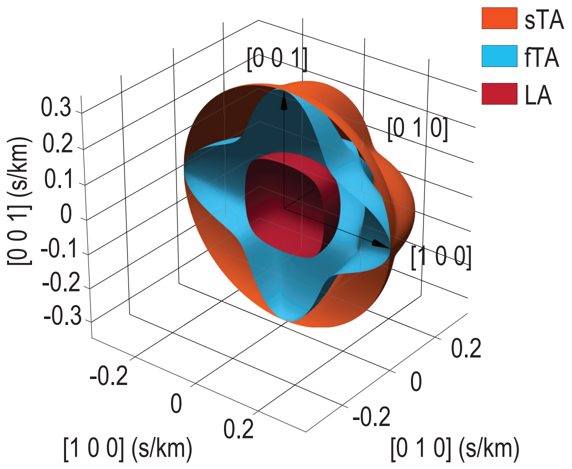

Figure 5: 3-D slowness surfaces of the three possible acoustic quasi-modes to propagate in CeO_2. Half of the polar angles are represented to obtain the cross-sectional view. (colors needed)

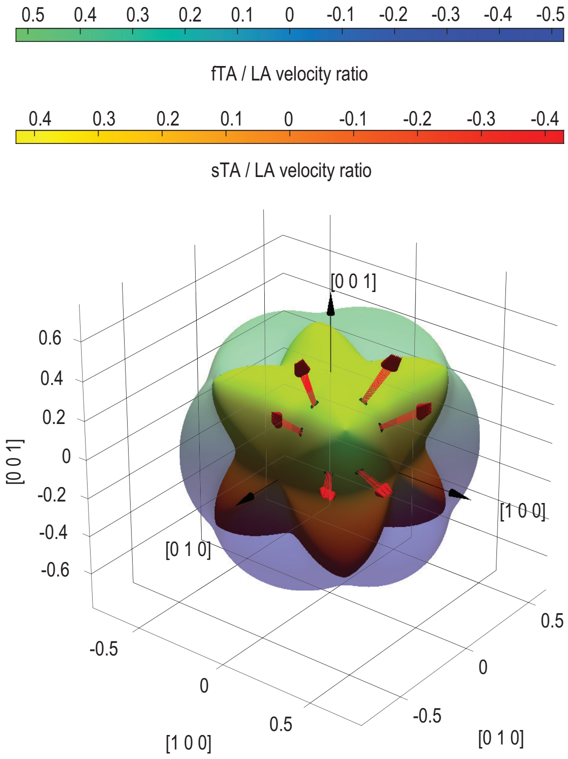

Figure 6: 3-D surfaces of shear velocity ratio to LA. The fTA/LA velocity ratio surface is transparent for better visualization of the sTA/LA one. The possible orientations of the grain 1A, in which the 3 acoustic quasi-modes are monitored, are represented by red arrows (with black circle basis) in 1/8 of the surface. Due to symmetries in the cubic crystalline structure, 6 orientations with common velocity ratio are obtained. (colors needed)

Table 3: Ceria's optical refractive index estimation.

Acoustic mode	Frequency (GHz)	Ratio (to LA)	Associated v ($\mathrm{m\,s^{-1}}$)	Deduced n
LA	61.65 ± 0.47	/	$6\,958 \pm 52$	2.37 ± 0.04
sTA	30.37 ± 0.23	0.4925 ± 0.008	$3\,428 \pm 33$	2.37 ± 0.03
fTA	40.15 ± 0.3	0.6515 ± 0.01	$4\,537 \pm 97$	2.37 ± 0.07

Supplementary materials on histogram segmentation inspired by Otsu's work for grains identification

Originally, Otsu's segmentation method [75] was used in image processing to separate foreground and background using the grayscale of an image.

Each pixel of the image is converted into an intensity value between one and 256 and then the histogram repartition of the intensity is plotted. To separate the background pixels from the foreground ones, a threshold in the [1, 256] intensity scale is obtained automatically by finding the location of the maximum value of the variance between the histogram's bins. This segmentation of the histogram, between the pixels

that contribute to the foreground and the ones that contribute to the background, splits the histogram in two equivalent parts with one threshold. Inspired by this method, the histogram repartition of the voxels, from the 4-D “sliced” output of the slicing post-processing part (Sec. 2.4), is plotted for each acoustic quasi-mode. The bin number and frequency edges are defined by the frequency resolution of the wavelet synchro-squeezed transform, thus defined by the wavelet filter bank used to compute the continuous wavelet transform prior to synchrosqueezing. Such parameter is controlled by the "number of voices per octave" defined in MATLAB’s documentation on the continuous wavelet transform. In our specific case, the aim is to locate the frequency intervals containing high occurrence bins. A grain is defined by a volume that contains a considerable amount of voxels having the same BF.

First, a threshold is defined as 5% of the total voxel amount in the histogram (solid red horizontal curve in Fig. 7), and the local maxima (red arrows in Fig. 7) are obtained by looking for peaks in the curve passing through all the bins (black solid curve in Fig. 7) having a prominence higher than half the threshold. Depending on the distribution of the bins surrounding the local maxima, two scenarios are possible. First, if the occupancies of the two surrounding bins are below the threshold, the BF interval defining this grain include the three bins. This case can be observed in the two high BF intervals in Fig. 7(a), and the low BF interval in Fig. 7(c). Secondly, if the surrounding bins have an occupancy above the 5% threshold, then closer attention needs to be given to the voxels repartition. If only one neighbouring bin is higher than the threshold, then the BF interval is shifted compared to the interval centred on the local maxima, along the frequency axis, in a direction towards the neighbouring bin with occupancy higher than the threshold. Such a situation is depicted by the low frequency BF interval in Fig. 7(a). Finally, if all the occupancies of all the neighbouring bins are above the threshold, the gradient of the occupancy curve is computed and is used to locally separate the two highest bins, and split in two BF intervals, hence in two grains, like in the case of the fTA mode depicted in Fig. 7(b). The final BF intervals defining the grains of our ceria scan are depicted by vertical dashed green lines in Figs. 7, and the associated values are gathered in table 4.

Each bin with occupancy above the threshold value could have been treated separately but would have finally appeared, from the alphashape output, as enveloping each other like the different layers of an onion. This could be justified by the fact that, when the BF is estimated close to a boundary between two grains, it could be slightly shifted due to the finite width of the window used for processing.

Table 4: Ceria’s grain intervals determined.

LA mode	Frequency (GHz)
LA 1	[61.19 ; 63.05]
LA 2	[64.91 ; 66.77]
LA 3	[67.70 ; 69.56]
**fTA mode**	**Frequency (GHz)**
fTA 1	[38.5 ; 40.18]
fTA 2	[40.18 ; 41.30]
**TA mode**	**Frequency (GHz)**
TA 1	[29.76 ; 30.70]
TA 2	[31.64 ; 33.05]
TA 3	[33.05 ; 33.99]

Supplementary materials on the depth of imaging limitations

Grains 3-D imaging results obtained in Fig. 3 appear to be imaged down to a depth of about 8 µm deep in the best cases. This limitation of the depth of imaging can be attributed to the coherence length of the probe laser beam, rather than other physical mechanisms such as the acoustic wave attenuation or the probe

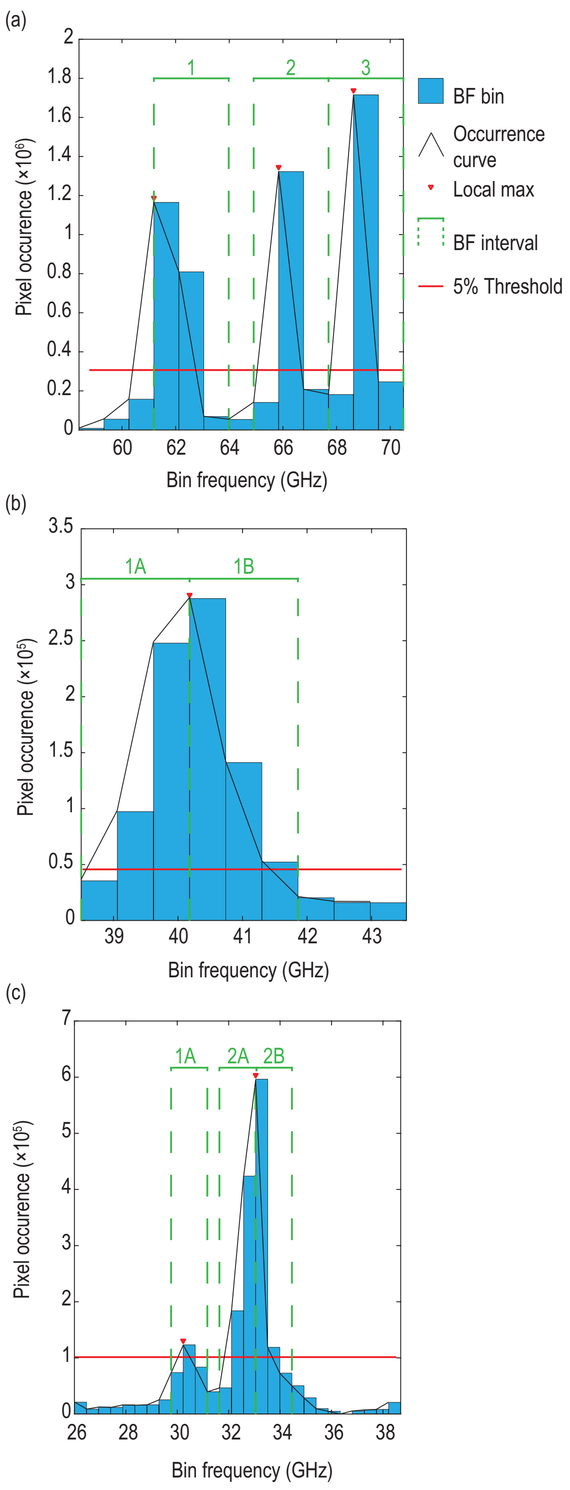

Figure 7: Histograms of the voxels repartition for each acoustic quasi mode: (a) LA, (b) fTA and (c) TA. (colors needed)

laser beam diffraction. To estimate the coherence length of the probe laser beam, one of the acoustic signals with the longest duration is selected (located at the position $x = 25.79$ µm and $y = 23.45$ µm of the scan), and a sinusoid with Gaussian and exponential dampings model is fitted to it. The model is given as:

$$\mathrm{A}\ \sin\left(2\pi f_{\mathrm{B}}^{\mathrm{LA}}\mathrm{t}+\phi\right)\ \exp\left(-2\left(\frac{\mathrm{t}}{\tau_{\mathrm{c}}}\right)^{2}\right)\ \exp\left(-\alpha\mathrm{t}\right), \tag{3}$$

with A the amplitude, $f_{\mathrm{B}}^{\mathrm{LA}} = 61.52$ GHz the BF of the LA mode obtained from the Fourier transform of the full acoustic signal, ϕ the phase, τ_{c} the coherence time of the probe laser pulse and α the acoustic absorption coefficient, of the signal.

Estimation of the coherence time from the fitting gives $\tau_{\mathrm{c}} = 1.16 \pm 0.012$ ns, the uncertainty being estimated with the 95% confidence interval of the fitting. Such a value can be checked from the relation between the coherence time and the duration of the probe laser pulse τ_{probe}:

$$\tau_{\mathrm{probe}} = \frac{2v_{LA}\tau_{\mathrm{c}}}{\mathrm{c}_0/n}, \tag{4}$$

with $v_{\mathrm{LA}} = (f_B^{LA}\lambda)/(2n) = 6\ 944$ m.s^{-1} the LA mode velocity, deduced from Eq. 1, and c_0 the speed of light

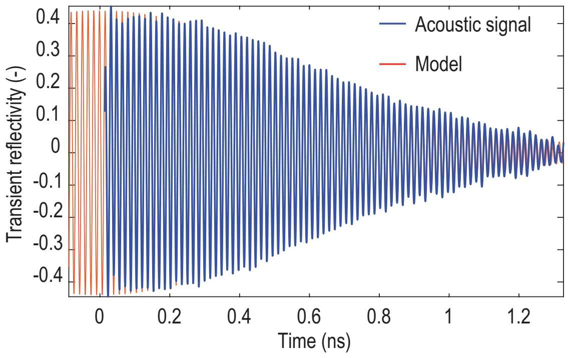

Figure 8: Comparison between the acoustic contribution to the transient reflectivity signal measured by the photodiode, located at the position $x = 25.79$ μm and $y = 23.45$ μm of the scan, and the Gaussian damping model signal obtained by fitting. (colors needed)

in vacuum. The probe laser pulses duration is thus estimated as $\tau_{\text{probe}} = 127 \pm 2$ fs, close enough to the laser datasheet $\tau_{\text{probe}} = 130$ fs. Finally to estimate the coherence penetration depth, one can either estimate the product of the coherence time τ_{c} by the LA mode velocity v_{LA}, giving $L_{\text{c}}^{\text{probe}} = 8.05 \pm 0.08$ µm, or use the estimated duration of the probe laser beam τ_{probe} with:

$$L_{\text{c}}^{\text{probe}} = \frac{(\text{c}_0/n)\tau_{\text{probe}}}{2}. \tag{5}$$

From this second method, the estimated coherence penetration depth is evaluated as $L_{\text{c}}^{\text{probe}} = 8.04 \pm 0.13$ µm, close to the previous estimate and in good agreement with the deepest observed 3-D images of the grain in the main text. The obtained fit is not improved by including the acoustic absorption since the fitted value for α is going to zero when not limited with a lower physical bound equal to 0. Hence the limiting factor for the depth of imaging arise from the penetration depth of the probe laser beam coherence.

REFERENCE

[81] D. Royer, E. Dieulesaint, Elastic Waves in Solids I: Free and Guided Propagation, Springer Science & Business Media, 1999.
